# Photocatalytical Antibacterial Activity of Mixed-Phase TiO_2_ Nanocomposite Thin Films against *Aggregatibacter actinomycetemcomitans*

**DOI:** 10.1155/2015/705871

**Published:** 2015-10-21

**Authors:** Sinem Yeniyol, Ilven Mutlu, Zhiming He, Behiye Yüksel, Robert Joseph Boylan, Mustafa Ürgen, Zihni Cüneyt Karabuda, Cansu Basegmez, John Lawrence Ricci

**Affiliations:** ^1^Department of Oral Implantology, Faculty of Dentistry, Istanbul University, 34093 Istanbul, Turkey; ^2^Metallurgical and Materials Engineering Department, Faculty of Engineering, Istanbul University, Avcilar, 34320 Istanbul, Turkey; ^3^Department of Basic Science and Craniofacial Biology, New York University College of Dentistry, New York, NY 10010, USA; ^4^Department of Mechanical Engineering, Istanbul Aydın University, 34668 Istanbul, Turkey; ^5^Department of Metallurgical and Materials Engineering, Faculty of Chemical and Metallurgical Engineering, Istanbul Technical University, 34469 Istanbul, Turkey; ^6^Department of Biomaterials and Biomimetics, New York University College of Dentistry, New York, NY 10010, USA

## Abstract

Mixed-phase TiO_2_ nanocomposite thin films consisting of anatase and rutile prepared on commercially pure Ti sheets via the electrochemical anodization and annealing treatments were investigated in terms of their photocatalytic activity for antibacterial use around dental implants. The resulting films were characterized by scanning electron microscopy (SEM), and X-ray diffraction (XRD). The topology was assessed by White Light Optical Profiling (WLOP) in the Vertical Scanning Interferometer (VSI) mode. Representative height descriptive parameters of roughness *R*_*a*_ and *R*_*z*_ were calculated. The photocatalytic activity of the resulting TiO_2_ films was evaluated by the photodegradation of Rhodamine B (RhB) dye solution. The antibacterial ability of the photocatalyst was examined by  *Aggregatibacter actinomycetemcomitans* suspensions in a colony-forming assay. XRD showed that anatase/rutile mixed-phase TiO_2_ thin films were predominantly in anatase and rutile that were 54.6 wt% and 41.9 wt%, respectively. Craters (2–5 *µ*m) and protruding hills (10–50 *µ*m) on Ti substrates were produced after electrochemical anodization with higher *R*_*a*_ and *R*_*z*_ surface roughness values. Anatase/rutile mixed-phase TiO_2_ thin films showed 26% photocatalytic decolorization toward RhB dye solution. The number of colonizing bacteria on anatase/rutile mixed-phase TiO_2_ thin films was decreased significantly *in vitro*. The photocatalyst was effective against *A. actinomycetemcomitans* colonization.

## 1. Introduction

Since the discovery of the photocatalytic oxidation for water on titanium dioxide (TiO_2_) electrode in 1972 by Fujishima and Honda [1], TiO_2_ is believed to be the most promising photocatalyst owing to its superior photoreactivity, nontoxicity, chemical stability, low price, and repeated use without substantial loss of catalytic activity [2–4]. Photocatalytic water splitting has been the focus of interest for TiO_2_-based photochemistry [5]. The fundamental mechanism underlying the photocatalytic killing has not been well established yet [6]. The photocatalytic activity of TiO_2_ is significantly influenced by various parameters including crystalline structure, impurities, surface area, and density of surface hydroxyl groups [7]. However, the most significant factor is its crystalline structure. TiO_2_ is usually used as a photocatalyst in two crystal structures: rutile and anatase. Anatase generally has a larger band gap (3.2 eV) than rutile (3.0 eV) with much higher photocatalytic activity [2]. The generation of electron-hole pairs is detrimental to this photocatalytic efficiency of the photocatalyst. Upon excitation by light with energy equal to or higher than the band gap energy, the photon energy generates electrons excited from the valence band to the conduction band and holes at the valance band on the TiO_2_ surface. The hole in the valance band can react with H_2_O or hydroxide ions adsorbed on the surface to produce hydroxyl radicals, and the electron in the conduction band can reduce O_2_ to produce superoxide ions (O_2_^−^). Both holes and OH^−^ are extremely reactive with contacting organic compounds [6, 8]. It was stated that these crystalline phases of TiO_2_, consisting of mixed rutile and anatase with an appropriate ratio, enhanced the photocatalytic properties [9]. For this reason, many studies commonly used the conventional mixed-phase photocatalyst powder Degussa P25 (Degussa Chemical Company, Teterboro, New Jersey, USA) consisting of anatase 80% and rutile 20% [2, 6, 10]. However, continuously stirring during the reaction process and their separation and recovery after reaction are their disadvantages. Recently, the use of immobilized thin TiO_2_ film coatings on substrates forming photocatalytical surfaces makes it possible to overcome these disadvantages [6, 11]. Studies have been focused on the development of immobilized TiO_2_ photocatalysts for their oxidative degradation of organic compounds as well as microorganisms [4]. Anatase/rutile mixed-phase TiO_2_ were synthesized by various methods including sol-gel method [12], sputtering [13], chemical vapour deposition [14, 15], atomic layer deposition [16], plasma immersion ion implantation [17], cathodic arc deposition [18], and anodization [19]. Among these methods, anodization is a cost-effective and simple technique that allows controlled anatase/rutile mixed-phase TiO_2_ formation on the substrates by means of altering the experimental conditions. To induce the TiO_2_ phases, generally an annealing treatment of as-prepared film at above 450°C is necessary.

Various photocatalytic activity studies have been conducted to develop antibacterial effect. Most of the studies have tested the photocatalytic activity on* E. coli* [2, 6, 20]. Kim et al. [21] have used food-borne pathogenic bacteria,* S. choleraesuis* subsp.,* V. parahaemolyticus*, and* L. monocytogenes*, and established a batch type photocatalytic reactor in order to investigate the bactericidal effect with various near-ultraviolet (UV) irradiation time and TiO_2_ concentrations. Lee et al. [22] had modified a defense mechanism to nullify* B. anthracis* in case of a probable microbial attack using the photocatalyst technology. The antibacterial efficiency of long wave UV-irradiated TiO_2_ thin films as well as the ultrastructural damage on bacterial cells was evaluated using* P. aeruginosa* as a model by Amézaga-Madrid et al. [23]. The most common and significant complication of dental implants is peri-implantitis which is closely related to the colonization of peri-implantitis-associated bacteria. Among these,* Aggregatibacter actinomycetemcomitans* is a gram-negative, nonmotile coccobacillus that colonizes the human oral cavity that jeopardizes the host's defense mechanism leading to peri-implantitis [24]. It is therefore most important to develop implant surfaces with antibacterial properties for maintaining plaque-free surfaces on titanium dental implants' transmucosal components exposed to the oral cavity. With the use of an appropriate anodization technique, universal control of the required surface properties resulting in reproducible photocatalytic anatase/rutile mixed-phase TiO_2_ thin films on almost any transmucosal component of a dental implant with antibacterial properties can be developed.

The present study is therefore designed to develop anatase/rutile mixed-phase TiO_2_ thin films by electrochemical anodization and annealing treatments in order to investigate their influence on the photocatalytic degradation of RhB dye solution and the colonization of peri-implantitis-associated bacteria* Aggregatibacter actinomycetemcomitans* as an index of antibacterial photocatalytic activity* in vitro*.

## 2. Materials and Methods

### 2.1. Preparation of Samples

Commercially pure titanium (cpTi) (ASTM B265-02) sheets in squares (10 × 10 × 1 mm) were used as substrates for the experiments. The surfaces of the specimens were prepared by standard metallographical techniques. These sheets were ultrasonically cleaned in acetone, distilled water, and methanol, respectively. These untreated cpTi sheets were named as* Group Ti*. The electrochemical anodization was employed to form TiO_2_ thin films on the cpTi sheets. Anodization voltage was performed under 40 V and each Ti surface (anode) was electrochemically anodized in 0.1 M KOH electrolyte for 3 minutes at 20°C [25]. Stainless steel was used as the counter electrode. In order to convert the amorphous TiO_2_ thin films into the crystallized TiO_2_ thin films, sheets were annealed at 550°C in air for 1 h after anodization treatment [26]. These sheets containing mixed-phase TiO_2_ thin films consisting of anatase and rutile were named as* Group AR*.

### 2.2. Artificial Saliva Preparation

Artificial saliva solution was prepared from chemicals supplied by Merck, Germany [27, 28]. Amounts of reagents are 0.40 g/L NaCl, 0.79 g/L CaCl_2_ H_2_O, 0.40 g/L KCl, 0.005 g/L Na_2_S 9H_2_0, 0.78 g/L NaH_2_PO_4_ H_2_O, and 0.35 g/L Urea-CO (NH_2_). The pH of the artificial saliva solution was 6.30. The pH value of the artificial saliva solution was measured and monitored using a pH meter (WTW, InoLab 720, Germany).

### 2.3. Electrochemical Corrosion Study

Electrochemical corrosion studies were carried out in the artificial saliva solution using a potentiostat (Interface 1000 Potentiostat/Galvanostat/ZRA, Gamry Instruments Inc., USA) controlled by a personal computer. Volume of glass corrosion test cell was 1000 mL. A conventional three-electrode system with high-density graphite rod as a counter electrode, a saturated calomel electrode (SCE) as reference electrode, and specimen as a working electrode was used. Data acquisition was carried out through a computer software (Framework, Version 6.04, Gamry Instruments, USA), while data analysis was carried out by Echem Analyst Software, Version 6.04, Gamry Instruments, USA. Specimens were prepared by mounting into epoxy resin. So, only square sheet surface of the specimens was exposed to the artificial saliva solution. The specimens were connected to a copper wire. All experiments were carried out at room temperature.

Open circuit potential (OCP) of the specimens was measured before carrying out the electrochemical corrosion experiments. OCP level was measured for durations of 2 to 3 hours, until the OCP was stabilized.

Tafel curves were obtained by polarizing the specimens from −250 mV to +250 mV (versus SCE), with respect to the OCP, at scan rate of 1.0 mV/s. Tafel slopes were obtained from Tafel extrapolation analyses.

Cyclic polarization tests were carried out from −500 mV (versus SCE) to apex potential and to final potential, which was 0 mV (versus SCE). Forward and reverse polarization scan rates were 5 and 2.5 mV/s, respectively. Cyclic (forward and reverse) polarization technique was used to evaluate tendency to localized corrosion (pitting) in corrosive environment. Considerable hysteresis between the forward and reverse polarization sweeps is an indication of the pit formation.

### 2.4. Surface Characterization

#### 2.4.1. White Light Interference Microscopy

The surface topologies of the sheets were investigated with the White Light Optical Profiling (WLOP) Wyko-NT1100 (Veeco Instruments Inc., Plainview, NY, USA) at VSI (Vertical Scanning Interferometer) mode, which is a noncontact optical profiling system that provides high vertical resolution. Two height descriptive parameters of roughness as *R*_*a*_ and *R*_*z*_ were used to quantify the surface roughness, where *R*_*a*_ is the arithmetical mean roughness value from the profile mean along a defined sampling line, and *R*_*z*_ is named as ten-point mean roughness which is the mean of maximum peak-to-valley height of 5 consecutive sections of the sampling line.

#### 2.4.2. Scanning Electron Microscopy

The surface morphologies of the sheets were observed using a scanning electron microscope (SEM; JSM5410, JEOL, Tokyo, Japan) at a 10 kV acceleration voltage and magnifications of ×500 and ×3500.

#### 2.4.3. X-Ray Diffraction

The structure and phase of the Group Ti were monitored by utilizing a Philips PW 3710 grazing incidence X-ray diffractometer with a CuK*α* radiation (scan range 20° to 80°). A scan rate of 0.02°/sec was used with a grazing incidence of 0.5° for the Ti structure. The structure and phase of the Group AR were monitored by utilizing a Panalytical diffractometer (Phillips, Holland) using X-ray diffraction data collected in the reflection Bragg-Brentano geometry with a CuK*α* radiation under an applied voltage of 45 kV and a current of 40 mA. A scan rate of 0.03°/sec with a grazing incidence of 0.45° was used for the Ti and TiO_2_ structure. The scanning data were recorded in the 2*θ* range of 20–73°. The phase contents of rutile and anatase (%*W*_A_) and rutile (%*W*_R_) for the anatase/rutile mixed-phase TiO_2_ thin films at Group AR were estimated by utilizing the obtained patterns to determine weight percentage of the anatase phase and rutile phase TiO_2_ using Spurr-Myers' equations [29] provided as in the following:

(1) %WR=11+0.884×IA/IR×100,%WA=11+1.26×IR/IA×100,

where *I*_R_ and *I*_A_ are the peaks areas in counts per second (c.p.s.) for the sharpest peaks for the (004) crystal plane of anatase TiO_2_ and (110) crystal plane of rutile phase TiO_2_, respectively. The numbers 0.884 and 1.26 are scattering coefficients [30, 31].

### 2.5. Photocatalytic Activity Evaluation

The photoactivity of the anatase/rutile mixed-phase TiO_2_ thin films was evaluated by studying their effect on the photodegradation of RhB dye solution (item # R6626, Sigma-Aldrich). RhB is a dye molecule, with a maximum absorption wavelength of 554 nm, that reacts with photogenerated oxyradicals and its loss may be monitored to evaluate the photocatalytic activity of the synthesized TiO_2_ samples using a UV-visible spectroscopy. A 30 W UV lamp irradiating a wavelength of 254 nm was used as the irradiation source. Sheets were irradiated in the perpendicular direction at a distance of 2 cm measured from the UV source to the initial sample surface. 5 mL of aqueous RhB dye solution was placed in each chamber to carry out the experiments with a starting concentration of 0.5 mg/L. RhB dye solution was pipetted onto each of the glass chambers that contained sheets from both experimental groups. Empty glass chambers served as the controls in this photocatalytic activity evaluation assay and were named as* Group C*. The decomposition of RhB dye was monitored by measuring the absorbency at 554 nm (*λ*max) by a UV-visible (UV-Vis) spectrophotometer (Perken Elmer Lambda 9 Spectrometer), and the degradation rate (%) was evaluated by the following equation [32]:

(2) $$\begin{array}{c} D=\frac{C_{t}}{C_{0}}\times 100\%, \end{array}$$

where *D* is degradation rate and *C*_0_ and *C*_*t*_ are the concentrations of the RhB dye solution at UV irradiation times 0 and *t*, respectively. To compare the rate of RhB dye photodegradation, the absorption spectra of each sample were recorded at timed intervals, up to 2 h (15 min, 30 min, 45 min, 60 min, 75 min, 90 min, 105 min, and 120 min). All the experiments were conducted thrice, and mean values were used as the final result.

### 2.6. Colony-Forming Assay for Antibacterial Effect

The tests were performed using* A. actinomycetemcomitans* (ATCC 43718; ATCC, Rockville MD, USA). Bacteria cells were cultured in brain heart infusion (BHI) broth (Thermo Scientific Remel, Lenexa, KS, USA) overnight at 37°C. Based on our pilot studies, the bacteria were grown to mid-log phase, centrifuged, and resuspended in trypticase soy broth to an optical density of approximately 0.40 at the wavelength 600 nm. Sheets from both experimental groups (*n* = 78 each) were placed into individual wells of the sterile 24-well culture plates with their modified surfaces placed facing upwards and bacteria cells were pipetted onto these sheets. Bacteria grown on culture plates' well bottoms were used as controls in this antibacterial assay and were named as* Group D* (*n* = 78). The culture plates were covered by their lids to prevent medium evaporation. Half of each group (*n* = 36 each) was exposed to UV irradiation (*as UV (+) condition*) (254 nm) while being incubated in an anaerobic environment (Modular Atmosphere Controlled System, DW Scientific, Shipley, Yorkshire, UK) at 37°C for 2 h, whereas the other halves (*n* = 36 each) were kept in a black box avoiding UV light penetration (*as UV (−) condition*) at the same experimental conditions. The supernatant fluid from each well was appropriately diluted and plated on TSBN media (personal communication, S. S. Socransky, Forsyth Institute, Cambridge, MA, USA) and incubated anaerobically for 4 days at 37°C and the number of colonies (colony-forming unit: CFU) was counted. TSBN was prepared as described elsewhere [33]. Antibacterial activity was expressed as the ratio of CFUs on each plate to those on the control group.

The power analysis was performed with PS Program (Power and Sample Size Program: http://biostat.mc.vanderbilt.edu/wiki/Main/PowerSampleSize) [34]. A* pre hoc* power analysis at 80% power, *α* = 0.05, Δ: 1.1, and SD: 1.5, was performed, and it was determined that a minimum of 30 sheets in each group were necessary when comparing Group AR, Group Ti, and Group D.

### 2.7. Statistical Analysis

All data were analyzed with the statistical package for social sciences, 22.0 (SPSS for Windows; SPSS Inc., Chicago, Illinois, USA). The normality test of Shapiro-Wilks was applied and the data were found normally distributed. Interactions between parameters were defined using the two-way analysis of variance (ANOVA) followed by subsequent one-way ANOVA and Student's *t*-test. If one-way ANOVA suggested a significant difference between means among the groups, when equal variances could be assumed,* post hoc* testing was done using the Tukey HSD test; otherwise, Tamhane's *T*2 test for those with unequal variances was used. When the *p* value was less than 0.05, the statistical test was determined as significant. Data were expressed as mean ± standard deviation.

## 3. Results

### 3.1. Electrochemical Corrosion Study

#### 3.1.1. Open Circuit Potential

**Figure 1:**
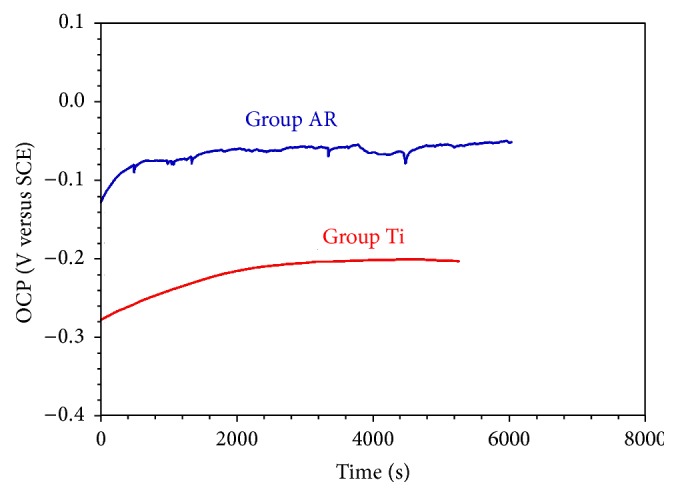
Open circuit potential (OCP) curves of the Group AR and Group Ti specimens.

Open circuit potential (OCP) is the potential at which an alloy is in equilibrium with the environment. High OCP value means that the material is stable in a certain corrosive environment.  Figure 1 shows the variation of the OCP level in the Group AR and Group Ti specimens. As shown in Figure 1, Group AR specimen showed higher OCP values than the Group Ti specimen, which means Group AR specimen has higher corrosion resistance than the Group Ti specimen.

#### 3.1.2. DC Corrosion Tests

**Figure 2:**
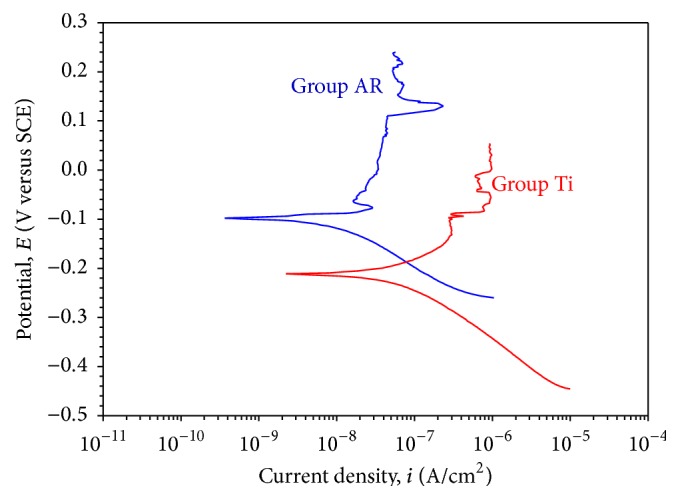
Tafel curves of the Group AR and Group Ti specimens.

The potentiodynamic polarization curves (Tafel curves) were used to examine the electrochemical corrosion behaviour of the specimens.  Figure 2 shows the Tafel curves of the Group AR and Group Ti specimens.

As illustrated in Figure 1, Group AR specimens showed higher corrosion potential and lower corrosion current density values than the Group Ti specimens.

TiO_2_ coating (Group AR specimens) increased the corrosion potential and decreased the corrosion current density of the specimens.

#### 3.1.3. Cyclic Polarization Test

**Figure 3:**
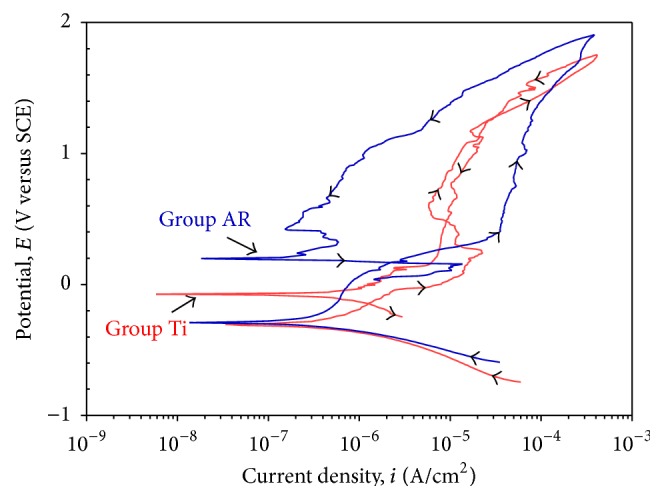
Cyclic polarization curves of the Group AR and Group Ti specimens.

Figure 3 shows the cyclic polarization curves of the Group AR and Group Ti specimens. As shown in Figure 3, Group AR specimen showed higher corrosion potential and lower corrosion current density values than the Group Ti specimen which means that the Group AR specimen has higher corrosion resistance than the Group Ti specimen. Cyclic polarization was used to qualitatively evaluate tendency to pitting (localized corrosion) in a corrosive environment. Hysteresis between forward and reverse sweeps during cyclic polarization is an indication of pit formation. As shown in Figure 3, hysteresis (loop) was not observed in the Group AR and Group Ti specimens.

### 3.2. Surface Characterization

#### 3.2.1. White Light Interference Microscopy

**Figure 4:**
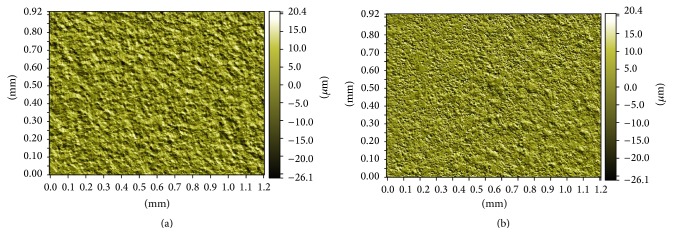
Representative WLOP images of (a) Group Ti, and (b) Group AR at ×5.1 magnification for 1 × 1.2 mm^2^ areas.

Quantitative roughness parameters *R*_*a*_ and *R*_*z*_ obtained from WLOP analysis for 1 × 1.2 mm^2^ areas for the Groups Ti and AR are shown in Table 1. Three-dimensional images of the surface topography of the Groups Ti and AR at ×5.1 magnification are shown in Figure 4. *R*_*a*_ and *R*_*z*_ surface roughness parameters values obtained from the Group AR quantitatively presented higher height descriptive parameters of roughness (Table 1).

#### 3.2.2. Scanning Electron Microscopy

**Figure 5:**
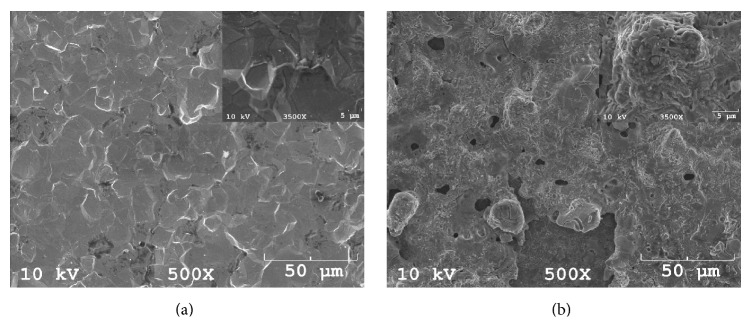
Representative top-view SEM micrographs of the (a) Group Ti, and (b) Group AR.

Figure 5 shows surface SEM images of sheets of cpTi (Group Ti) and the mixed-phase TiO_2_ thin film photocatalyst (Group AR). It is observed that cpTi surface has a flat texture and showed relatively a smooth appearance. The surface morphology of the photocatalyst was affected by the electrochemical anodization. Craters (2–5 *μ*m) and protruding hills (10–50 *μ*m) were observed in Group AR anodized in the KOH electrolyte, conferring a more pronounced increase of surface roughness compared to cpTi sheets in Group Ti.

#### 3.2.3. X-Ray Diffraction

**Figure 6:**
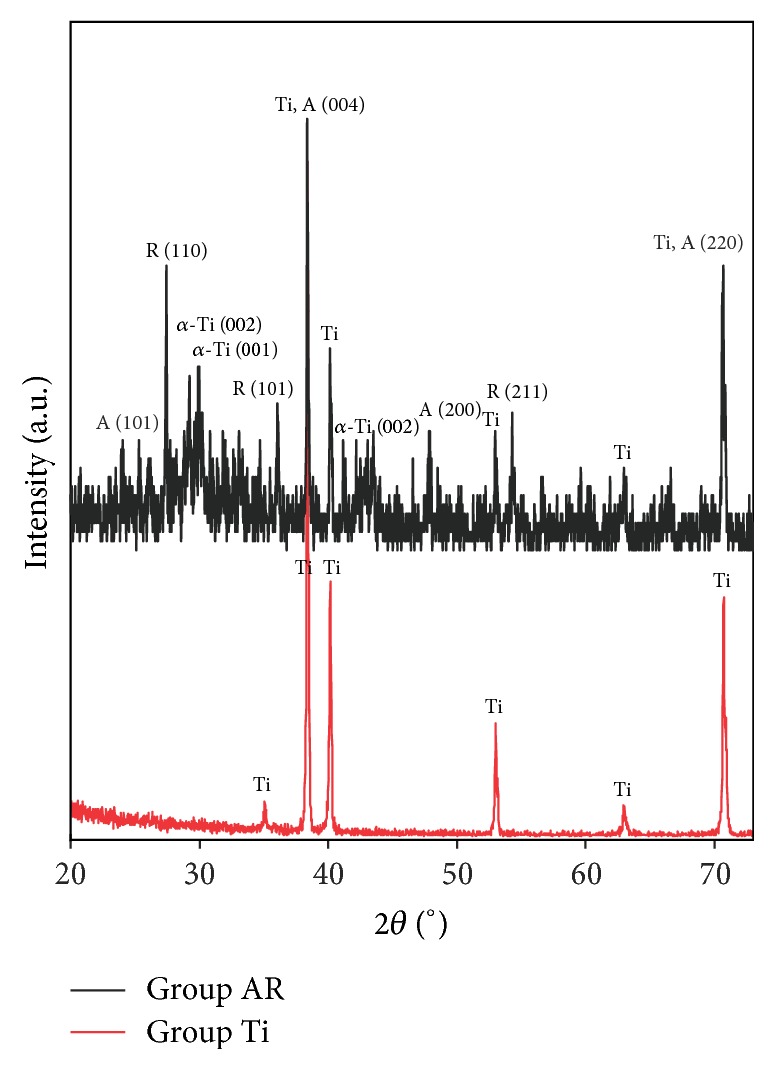
X-ray diffraction patterns of Group Ti: cpTi surface; Group AR: anatase/rutile mixed-phase TiO_2_ thin film surface (Ti: titanium; A: anatase; R: rutile).

The phase compositions of the obtained samples were characterized by XRD and the corresponding XRD patterns are shown in Figure 6. There was no anatase or rutile diffraction peaks observed in cpTi surfaces in Group Ti. After anodization treatment and annealing at 550°C for 1 h, the mixed-phase composition of the Group AR was confirmed by its XRD patterns in Figure 6, where two sharp peaks located at the 2*θ* values of 38.4° and 27.4°, which are attributed to the diffraction of the (004) crystal plane of anatase TiO_2_ and the (110) crystal plane of rutile phase TiO_2_, respectively, indicating the crystallinity of the structure. Other characteristic peaks of the different crystalline phases are also marked on the pattern (Ti: titanium; A: anatase; R: rutile). Anatase and rutile contents (weight percentages) in each sample were confirmed by using Spurr-Myers' equations (see (1)) and the corresponding results of anatase and rutile were calculated as 54.6 wt% and 41.9 wt%, respectively.

### 3.3. Photocatalytic Activity Evaluation

**Figure 7:**
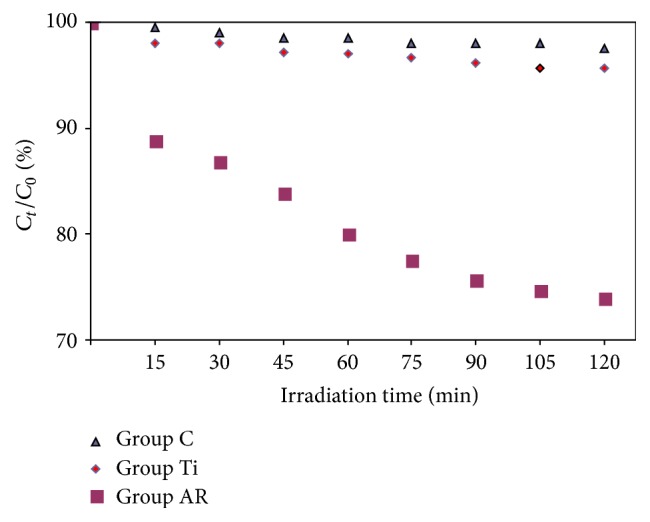
Photodegradation rate of RhB dye solution as a function of time under UV light irradiation on Group Ti: cpTi surface; Group AR: anatase/rutile mixed-phase TiO_2_ thin film surface; Group C: glass surface.

The photocatalytic performance of anatase/rutile mixed-phase TiO_2_ thin films in the degradation reaction of RhB dye solution was tested.  Figure 7 shows the photocatalytic activity of both anatase/rutile mixed-phase TiO_2_ thin films and cpTi surfaces under irradiation by UV light. The degradation of original RhB dye solution without photocatalyst was also plotted for comparison on glass as the control group. RhB dye concentration was decreased only by 4.4% and 2.4% (Group Ti and Group C, resp.) by the irradiation of ultraviolet for 120 min without the photocatalyst. This decomposition of RhB dye can be attributed to photolysis of RhB dye by ultraviolet. On the other hand, when the anatase/rutile mixed-phase TiO_2_ thin films were used as photocatalyst, 22% RhB dye was degraded in 60 min, and the photocatalytical activity of RhB dye was maintained up to 26% in 120 min.

### 3.4. Colony-Forming Assay for Antibacterial Effect

**Figure 8:**
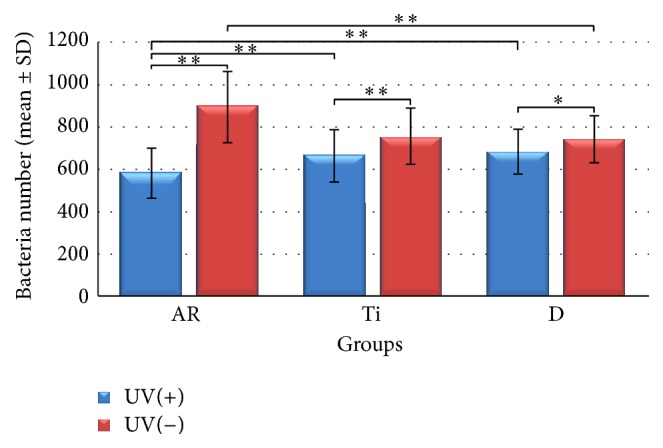
Descriptive analysis of adhesion of* A. Actinomycetemcomitans* on all groups tested (Group Ti: cpTi surface; Group AR: anatase/rutile mixed-phase TiO_2_ thin film surface; Group D: culture plates' well bottoms). Data are presented as the mean ± SD (*n* = 39) for all bars. Results were analyzed using the two-way analysis of variance (ANOVA) followed by subsequent one-way ANOVA and Student's *t*-test. If one-way ANOVA suggested a significant difference between means among the groups,* post hoc* analyses were performed using Tukey HSD test, and Tamhane's *T*2 test (^*∗*^ *p* < 0.05, and ^*∗∗*^ *p* < 0.01).

UV irradiation had led to a significant decrease in the number of bacteria for all groups (Groups Ar and Ti: *p* < 0.01; Group D: *p* < 0.05). The number of bacteria decreased significantly in Group AR after 120 min of UV irradiation compared with all the other groups (*p* < 0.01) while bacteria were still detected after 120 min in all groups. In both UV (+) and UV (−) conditions no significant difference was found between the Groups Ti and D. Group AR showed statistically increased number of bacteria in UV (−) conditions (Groups Ti and D, *p* < 0.01) (Figure 8).

## 4. Discussion

The aim of this study was to develop antibacterial transmucosal components of dental implants less prone to peri-implantitis-associated bacteria colonization. This objective was achieved via surface modification by electrochemical anodization and annealing treatments. This study showed that anatase/rutile mixed-phase TiO_2_ thin films inhibited adhesion of* A. actinomycetemcomitans*.

Based on previous studies, it is shown that surface roughness of the exposed transmucosal components is related with intraoral bacteria colonization over that surface [35]. In our subject, greater surface roughness can increase the total surface area, therefore creating more available active surface sites for reactions by higher photocatalytic reaction potential. By gaining a better understanding of the surface roughness and crystalline structure of different types of transmucosal dental implant components, practitioners will be better placed to utilize the most suitable part for any given prosthetic indication and to interpret the antibacterial clinical performance of these parts in relation to the patient needs and intraoral usage sites.

TiO_2_ is usually used as a photocatalyst in two crystal structures: rutile and anatase. A postannealing is required to crystallize amorphous TiO_2_ into anatase, rutile, or brookite structure [36]. In the present work, we aimed to form crystallized mixed-phase TiO_2_ thin films consisting of anatase and rutile by annealing the electrochemically anodized sheets at 550°C after electrochemical anodization. Anatase/rutile mixed-phase TiO_2_ thin films obtained by electrochemical anodization and annealing treatments were investigated by the photodegradation of RhB dye solution in order to prove photocatalytic activity. The rate of photodegradation was monitored by the decrease in the absorption value of the peak at 554 nm.  Figure 7 shows the photodegradation efficiency of RhB dye solution as a function of UV irradiation time for the Groups Ti and AR as well as glass as control. The decrease in the absorption spectra of the dye solution was monitored at regular intervals of time. Compared to the cpTi surfaces the anatase/rutile mixed-phase TiO_2_ thin films exhibited photocatalytic efficiency. After two hours of irradiation under UV light, the RhB dye solution was degraded 26% by the anatase/rutile mixed-phase TiO_2_ thin films. In order to elucidate and examine the above results, the crystallinity of the samples was detected by XRD and is shown in Figure 6 presenting several dominant peaks of anatase and rutile phase after annealing process. They indicated two sharp diffraction peaks at 2*θ* = 38.4° and 27.4° that are identified to be (004) crystal plane of anatase TiO_2_ and the (110) crystal plane of rutile phase TiO_2_, respectively. Anatase and rutile contents (weight percentages) in each sample were calculated as 54.6 wt% and 41.9 wt%, respectively (Figure 6). Thus, electrochemical anodization treatment and annealing (550°C for 1 h) rendered a feasible and facile method to grow anatase and rutile phase TiO_2_ from cpTi. This photocatalytic efficiency obtained from the anatase/rutile mixed-phase TiO_2_ thin film containing about 54.6 wt% anatase phase and 41.9 wt% rutile phase was maintained up to 26% in 120 min. Our work illustrates that anatase/rutile mixed-phase TiO_2_ thin films show higher photodegradation efficiency than the cpTi surfaces under UV in our run confirming enhanced photoactivity of cpTi titanium by electrochemical anodization and annealing treatments. This increase at the photocatalytical activity can be attributed to the difference in band gap energy of anatase and rutile structures of mixed TiO_2_ films that were found to decrease the electron and hole recombination rate leading to an increase in the absorption of the surface organic species of the photocatalytic process [8]. Another explanation proposed by Hurum et al. (2003) is that, in mixed-phase TiO_2_ under the presence of rutile crystals, charges produced on rutile by visible irradiation are stabilized through electron transfer to lower energy anatase lattice trapping sites at the transition points between these two phases [37]. Subsequent electron transfer moves the electron from anatase trapping sites to surface trapping sites, further separating the electron/hole pairs process [8]. The efficiency of photocatalysts is determined by recombination rates. Thus, rutile reduces the charge recombination rate of anatase and acts as an antenna to extend the photoactivity into visible wavelengths [37].

Electrochemical corrosion test results indicated that the passive oxide coating on the Ti specimens enhanced the corrosion resistance. Group AR specimens showed higher corrosion potential and lower corrosion current density than the Group Ti specimens, which was attributed to its protective passive oxide coating (Figures 1–3).

Photocatalytic TiO_2_ coatings create contact-active surfaces that destroy the viability of the contacting microbes by photoenhanced formation of hydroxyl radicals [38]. This photocatalytic killing effect has been used in studies to eradicate bacteria around dental implants. Riley et al. (2005) considered if photoactive dental implants coated with nanostructured TiO_2_ would eradicate* E. coli* when illuminated with UV light. The photoactivity of dental implants was established by photoenhanced decomposition of RhB dye solution. Irradiation of dental implants with UV light was found to be a suitable treatment for peri-implantitis [39]. In their study, photocatalytic activity was questioned to find a solution for peri-implantitis. But, the bacterium used was not intraoral bacterium which would not be expected to mimic the oral environment of a dental implant. Suketa et al. (2005) reported the bactericidal effect of the TiO_2_ photocatalyst to be of great use for sterilizing the contaminated surface of dental implants.* Actinobacillus actinomycetemcomitans* and* Fusobacterium nucleatum* were chosen for their study as these bacteria are responsible for the induction of inflammation of the gingivae, destruction of the periodontal ligament and alveolar bone associated with periodontal disease. The viability of both types of bacteria on the photocatalytic TiO_2_ film was suppressed to less than 1% under UVA irradiation within 120 minutes [40]. Similarly, we chose* A. actinomycetemcomitans* for the colony-forming assay in our study has been implicated as it is the causative agent of several forms of severe periodontal disease [41]. UV irradiation had led to a significant decrease in the number of* A. actinomycetemcomitans* for all groups (Groups Ar and Ti: *p* < 0.01; Group D: *p* < 0.05) showing a killing effect of its own on all groups. Anatase/rutile mixed-phase TiO_2_ thin films in Group AR significantly decreased the adhesion of* A. actinomycetemcomitans* after 120 min of UV irradiation compared with all the other groups (*p* < 0.01) showing a statistically significant photocatalytical killing effect in addition to the UV irradiation's killing effect. Antibacterial effect against* A. actinomycetemcomitans* on anatase/rutile mixed-phase TiO_2_ thin films can be ascribed both to the UV irradiation and photocatalytical activity of Group AR containing crystal structures of anatase and rutile. But this combined killing effect was not able to kill all the bacteria on the surface of Group AR (Figure 8). In UV (−) conditions, Group AR showed statistically increased number of bacteria in UV (−) conditions (Groups Ti and D, *p* < 0.01) depending on its higher surface roughness values (Figure 4 and Table 1). cpTi surfaces in Group Ti were observed to be smooth and flat before electrochemical anodization (Figure 5(a)). Photocatalytically active anatase/rutile mixed-phase TiO_2_ thin films with surface features of craters (2–5 *μ*m) and protruding hills (10–50 *μ*m) on Ti substrates (Figure 5(b)) were produced after electrochemical anodization. This enhancement in the bacteria number may be mainly attributed to the higher surface roughness of the anatase/rutile mixed-phase TiO_2_ thin films allowing higher bacterial adhesion.

Photooxidation reaction of the terminal sulfhydryl group of CoA, which participates in many enzymatic reactions involved in the respiratory chain and fatty acid oxidations, is the reactive site of this molecule for the acyl transfer reactions and it is detrimental to cell viability. Nonselective actions of the highly oxidized species generated on the surface of the illuminated TiO_2_ are expected to oxidize the cell membrane [42]. In this regard, a thorough understanding of the photocatalytical effect on the cell killing mechanisms of the human gingival soft tissues around dental implants should be questioned in addition to its suggested killing effect on oral bacteria around dental implants.

## 5. Conclusions

Anatase/rutile mixed-phase TiO_2_ thin films fabricated by electrochemical anodization and annealing showed photocatalytic performance. The photocatalytic mechanism of TiO_2_ film with mixed structure and the relationship between the contents of anatase with rutile phase brought forth antibacterial activity of these films which may further be considered for the antibacterial improvement applications for the transmucosal components of the dental implants. The findings, however, have to be verified in clinical settings.

## Figures and Tables

**Table 1:** *R*_*a*_ (arithmetical mean roughness) and *R*_*z*_ (ten-point mean roughness) of the Groups Ti and AR determined by WLOP for 736 × 480 *µ*m^2^ areas. Data are presented as the mean ± SD (standard deviation). Values are in micrometers (*µ*m).

Groups	*R*_*a*_ (*µ*m) ± SD	*R*_*z*_ (*µ*m) ± SD
Ti	1.51 ± 0.02	12.76 ± 0.04
AR	4.08 ± 0.02	42.40 ± 0.27
